# Defect Detection in Wood Using Air-Coupled Ultrasonic Technique Based on Golay Code

**DOI:** 10.3390/s25103168

**Published:** 2025-05-17

**Authors:** Jun Wang, Tianyou Xu, Hongyan Zou

**Affiliations:** 1 College of Information Science and Technology, Nanjing Forestry University, Nanjing 210037, China; xutianyou@njfu.edu.cn; 2 College of Mechanical and Electronic Engineering, Nanjing Forestry University, Nanjing 210037, China; zouhy@njfu.edu.cn

**Keywords:** phase encoding, air-coupled ultrasound, non-destructive testing

## Abstract

Air-coupled ultrasound overcomes the limitations of traditional contact-based ultrasonic methods that rely on liquid couplants. Still, it faces challenges due to the acoustic impedance mismatch between air and wood, causing significant signal scattering and attenuation. This results in weak transmission signals contaminated by clutter and noise, compromising measurement accuracy. This study proposes a coded pulse air-coupled ultrasonic method for detecting defects in wood. The method utilizes Golay code complementary sequences (GCCSs) to generate excitation signals, with its feasibility validated through mathematical analysis and simulations. A-scan imaging was performed to analyze the differences in signal characteristics between defective and non-defective areas, while C-scan imaging facilitated a quantitative assessment of defects. Experimental results demonstrated that GCCS-enhanced signals improved the ultrasonic penetration and axial resolution compared to conventional multi-pulse excitation. The method effectively identified defects such as knots and pits, achieving a coincidence area of 85% and significantly enhancing the detection accuracy.

## 1. Introduction

Wood undergoes a biologically inherent defect generation (including knot formations and pit cavities) during biological maturation phases and industrial handling stages (harvesting, transportation, and machining), with these anomalies interrupting fiber alignment and inducing stress concentrations that irreversibly degrade both the mechanical strength and esthetic uniformity [1,2,3]. Non-destructive testing (NDT) technologies, when applied before wood processing, facilitate the detection of defects and the creation of rational cutting plans. This leads to an improved product quality, minimized material waste, and reduced safety risks [4,5]. Ultrasonic waves, characterized by strong directivity, high penetration capability, and versatility, are widely employed in wood defect detection [6,7,8]. Studies demonstrate that analyzing ultrasonic energy attenuation or time-of-flight variations during propagation through wood enables the accurate identification of defect types, locations, and dimensions [9,10]. However, conventional ultrasonic methods, whether using contact or immersion techniques, require liquid couplants between the transducer and the sample surface to enhance signal transmission. This, however, risks contaminating the material [11,12,13].

To avoid the pollution of wood, non-contact and non-destructive testing methods have gradually come into people’s vision, and it is very common to use machine vision technology to detect wood defects. Hittawe et al. employed machine vision technology to detect knots and classify them into transverse knots and non-transverse knots. Based on this classification, they calculated the knot depth ratio (KDR) using all identified transverse knots and subsequently improved the wood mechanical model with the KDR. The accuracy of their findings—including the knot quantity, spatial distribution, elastic modulus, and rupture modulus—was validated through a dataset containing 252 images [14]. Nurthohari et al. utilized a Histogram of Oriented Gradients (HOG) based on machine learning (ML) techniques to identify wood grain patterns and textures. They employed Support Vector Machines (SVMs) to classify images of five cedar species, achieving a recognition accuracy of up to 90% [15]. Ji et al. implemented a wood defect detection system by integrating deep learning-based predictive models with YOLO-embedded network architectures. This framework incorporates SIFT-feature-based image stitching, segmentation, and fusion techniques to enhance defect localization capabilities. Experimental results demonstrate that the system maintains a robust performance under complex conditions, achieving a detection accuracy of 90.37% even with significant background noise and interference factors [16]. Lukovic et al. developed a ResNet50-based residual network model trained on 15,000+ high-resolution images to predict the strength-related properties of Norway spruce (Picea abies) wood veneers through a color image analysis. The model achieved a coefficient of determination (R^2^) slightly exceeding 0.9, demonstrating a robust correlation between visual features and mechanical characteristics [17]. While machine vision technology achieves high-precision detection for wood surface defects, it faces critical blind spots in subsurface inspections. Due to restrictions on its external visual analysis, this method cannot identify internal structural flaws (e.g., hidden cracks or voids), significantly increasing the risk of associated safety hazards. Ultrasonic testing (UT), however, overcomes this limitation by leveraging material-penetrating waves to directly evaluate wood integrity.

Air-coupled ultrasound (ACU) uses air as the coupling medium, allowing for a non-contact inspection. This method avoids contaminating the sample and achieves a true non-destructive evaluation [18,19,20]. However, when employing air-coupled ultrasound for the non-destructive testing of wood, the detection capabilities and accuracy are frequently limited by the axial resolution and penetration depth of the ultrasonic waves. Axial resolution is the minimum distance that can be distinguished between two adjacent structures along the direction of the ultrasound beam. It is mainly determined by the pulse width and the center frequency of the ultrasonic signal. Narrower pulse widths and higher frequencies generally result in a higher axial resolution, thereby enhancing the detection clarity. The penetration depth, defined as the maximum distance ultrasonic waves can propagate in a medium, is inversely proportional to the frequency in high-acoustic-impedance solids such as wood. This relationship exists because higher frequencies undergo greater energy attenuation within dense materials. As a result, increasing the axial resolution through frequency optimization will inherently decrease the penetration depth. Thus, enhancing the axial resolution while preserving a sufficient penetration depth is crucial for achieving high detection accuracy in wood defect characterization.

Numerous studies have explored methods to maintain the ultrasonic penetration depth. Kazys et al. developed high-performance air-coupled ultrasonic transducers using PMN-32% PT crystals, which enhance the acoustic pressure output through optimized piezoelectric properties and vibration modes [21]. Zhang et al. used a piezoelectric transducer with a sandwich structure and a longitudinal vibration of a step-shaped ultrasonic transformer to excite the vibration of a metal ball, which improved the output power of the ultrasonic transducer [22]. Zhang et al. designed an air-coupled capacitive ultrasonic transducer array with MXenes as the sensitive material and found that this structure can significantly affect the sound pressure level of the transducer [23]. Yu et al. used multiple Helmholtz resonator holes and increased the output pressure of the ultrasonic transducer by 32.1% [24]. Fang et al. used anodically oxidized aluminum as the ultrasonic transducer matching layer to reduce the bidirectional insertion loss and improve the transducer output efficiency [25].

Reducing the ringing effect of the ultrasonic transducer can enhance the axial resolution of the ultrasonic wave without changing the center frequency. Kusano et al. improved the oscillation time of piezoelectric micro-machined ultrasonic transducers (PMUTs) by adjusting the polarization of the piezoelectric material [26,27]. Liu et al. reduced the ringing time of the piezoelectric micro-machined ultrasonic transducer (PMUT) by phase shifting [28]. Yang et al. proposed a new optimization scheme for the geometry of 1–3 piezoelectric materials to reduce acoustic crosstalk between the pulse–echo and frequency ultrasound [29]. Wu et al. discovered that a direct current bias voltage can effectively reduce the ringing time of piezoelectric micro-machined ultrasonic sensors based on aluminum nitride [30]. Wu et al. proposed a transmission-based attenuation suppression system that can reduce the oscillation time of the 115 kHz PMUT array by up to 93% [31]. Although these studies have enhanced the performance of ultrasonic transducers, they generally require significant modifications to the transducers’ internal structure or external circuitry. Such alterations not only increase hardware costs but also compromise their versatility across diverse application scenarios.

Encoding the excitation signal and decoding the received echoes through pulse compression techniques enables simultaneous improvements in the ultrasound penetration depth (via wide pulses) and axial resolution (via matched filtering) [32,33,34,35,36]. In 2010, Garcia-Rodriguez proposed a non-destructive testing system based on an air-coupled piezoelectric array that uses Golay sequences to encode the Lamb waves excited in thin materials, and the copper plate is used as the experimental object; the results show that compared with the traditional pulse transmission, the signal-to-noise ratio is improved by 21 dB [37]. In 2022, Tang et al. introduced phase encoding excitation and pulse compression technology in order to reduce the influence of the chromatic dispersion characteristics of waves, the signal attenuation, and other factors on the signal-to-noise ratio and realized the accurate evaluation of defects in the detected sample [38]. In 2024, Yang et al. used multivariate coding excitation (MCE) to excite the ultrasonic guided wave to monitor the rail, and it was found that the gain of the echo amplitude after the rail fracture and the amplitude difference (DIA) of the healthy rail increased significantly. Experimental results show that MCE compensates for the shortcomings of traditional excitation methods and improves the excitation energy and detection accuracy of the monitoring system [39]. In 2025, Germano et al. used a lead-free high-frequency array to detect 100 mm thick flat aluminum and found that compared with the conventional pulse excitation time-of-flight method (TFM), the time-of-flight method (TFM) based on the Golay code showed a better performance, and the signal-to-noise ratio, penetration depth, and image quality were more excellent [40]. Although coding excitation technology has been scaled up and implemented in industrial non-destructive testing, the current research focus remains largely on the detection of metallic materials. However, the potential of this technology in terms of the signal modulation strategy, noise suppression method, and quantitative characterization of defects has not been systematically explored for wood, a material with complex anisotropic characteristics. The field urgently needs to develop a new system of coded pulse ultrasound technology for wood to achieve the high precision non-destructive testing of defects.

This study discusses the application of a coded pulse technique in air-coupled ultrasonic testing for internal defects in wood. By employing a combination of Golay codes to encode the wide pulse excitation signal and decoding and compressing the received echo, the penetration depth and axial resolution of the ultrasonic wave can be enhanced while maintaining a constant output power of the ultrasonic transducer, ensuring the reliability of the detection results. Compared to non-encoded pulse excitation, the coded pulse technique significantly enhances the accuracy of ultrasonic detection for wood defects. In the detection of knots and pits in wood, it can accurately determine the types and sizes of defects, and the coincidence rate of its detection results with the actual defective area is 85%.

The following sections will sequentially introduce the system composition, experimental principles, scanning methods, and experimental procedures. First, the system architecture is presented using a block diagram, with a detailed explanation of the ultrasonic transmission method. Second, the principles of encoding and decoding eight-bit Golay complementary sequences are explained, accompanied by MATLAB R2022B simulation experiments. Third, physical images of the experimental platform are displayed, with particular emphasis on the motion system, scanning approaches, and imaging methodologies of the detection platform. Finally, defect detection tests are conducted on different wooden board specimens, followed by a comparative analysis of various inspection methods.

## 2. The Principle of Air-Coupled Ultrasonic Wood Defect Detection Based on the Golay Code

### 2.1. The Composition of Air-Coupled Ultrasonic Wood Defect Detection System Based on the Golay Code

**Figure 1:**
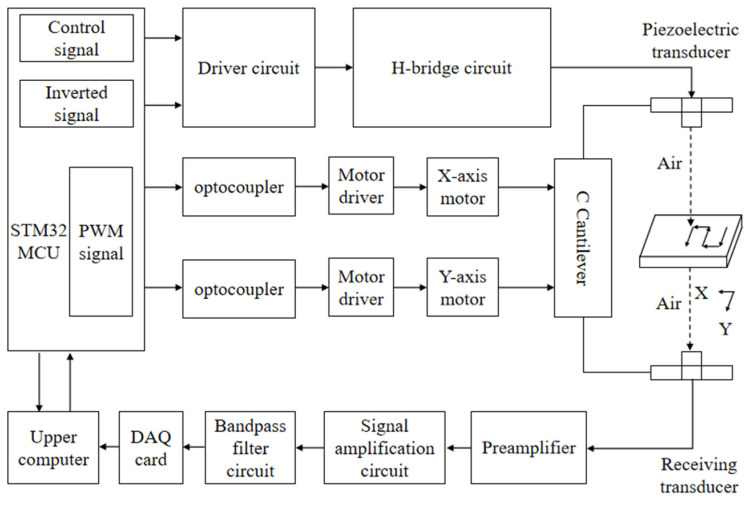
The block diagram of the air-coupled ultrasonic wood defect detection system based on the Golay code.

The air-coupled ultrasonic wood defect detection system, which utilizes the Golay code, is depicted in Figure 1. The experimental platform, constructed from an aluminum alloy, incorporates vertically aligned 75 kHz ultrasonic transducers. The STM32F103VET6 which produced by STMicroelectronics (Geneva, Switzerland) generates two unique eight-bit sequence signals, Sequence A and B, which are then processed by an H-bridge driver circuit to produce ±200 V Golay-coded excitation signals. The propagation of ultrasonic waves through the specimen is significantly attenuated due to scattering phenomena arising from the acoustic impedance mismatch at the air–sample interface. This results in a transmitted signal with weak energy, making it susceptible to interference from stray waves. Consequently, a Golay code transmission signal reception module has been designed to enhance the signal quality. Firstly, the preamplifier actively suppresses DC offset artifacts inherent in the Golay code transmission signal. Secondly, the transmitted signal is amplified by a gain amplifier, and the bandpass filter circuit removes the interference and noise therein. Finally, the Golay code transmission signal is transmitted to the upper computer for analysis and processing through the DAQ card. Once the upper computer transmits the scanning range and interval parameters of the C-scan to the lower computer controller, the STM32 controller directs the movement of the two-axis stepping motor by these instructions until the C-scan process is completed. Following the C-scan, the upper computer analyzes the changes in the key characteristic parameters of ultrasonic waves and converts them into colored pixel points, thus forming an RGB color map that displays the position of defects, so as to realize the visual positioning of wood defects.

### 2.2. Principle of Air-Coupled Ultrasonic Plate Testing

**Figure 2:**
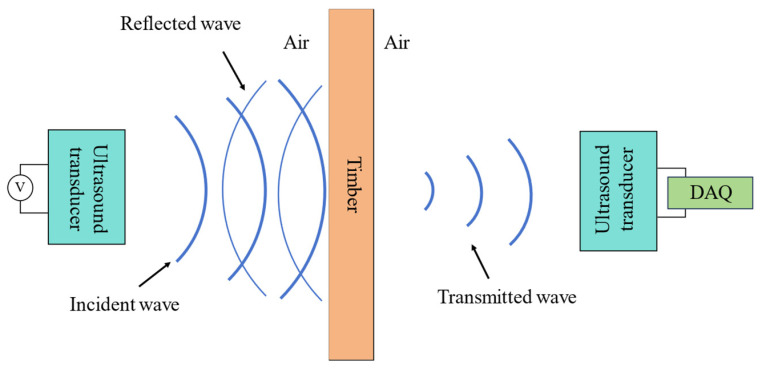
Schematic diagram of ultrasonic transmission method.

This study implemented a non-destructive evaluation of timber panels through the air-coupled ultrasonic technique. The ultrasonic transducer generated ultrasonic signals under the excitation of high-pressure pulse signals. The signal passes through the air and arrives at the interface between the wooden plate and the air. Due to the mismatch of the acoustic impedance between the two media, most of the sound energy is lost due to reflection. A minor proportion of the ultrasonic energy traverses the wooden panel, propagates through the intervening air medium, and is ultimately detected by the reception transducer (Figure 2).

Let Z_1_ represent the acoustic impedance of air and Z_2_ represent the acoustic impedance of the wood.

The sound pressure reflection coefficient is denoted by r, as shown in Equation (1):

(1) $$r=\frac{Z_{2}-Z_{1}}{Z_{2}+Z_{1}}$$

The sound pressure transmission coefficient is represented by t, as shown in Equation (2):

(2) $$t=\frac{2Z_{2}}{Z_{2}+Z_{1}}$$

The acoustic impedance of air media is significantly lower than that of liquids and solids, typically around 400 Rayls. In contrast, the acoustic impedance of solid media is generally higher, with wood having an impedance of approximately 1,600,000 Rayls. From Equations (1) and (2) above, it is evident that the sound pressure reflection rate is roughly 99%, while the sound pressure transmission rate is about 1%. Consequently, the ultrasonic signal transmitted through wood may become very weak or even vanish.

The optimization of the ultrasonic transmission through lignocellulosic media requires the precise adjustment of the actuating transducer’s excitation parameters to enhance the output energy. The number of excitation signal pulses is typically increased from a single pulse to multiple pulses to enhance the energy intensity of the ultrasonic signal. When employing multiple pulse excitation, although the signal’s penetration ability is improved, the length of the ultrasound signal also increases, which diminishes the signal’s resolution and the accuracy of the system detection. Consequently, coded ultrasound technology is employed to modulate the excitation waveform, and through the demodulation of the propagated signals, it can preserve the high-fidelity axial resolution while augmenting the penetration efficacy of the ultrasonic propagation.

### 2.3. Golay Coding Principle

Golay codes are a pair of equal-length complementary sequences generated by fixed rules and containing only the elements of “1” and “−1”, with sequence A (a_0_, a_1_, …, a_N_) and sequence B (b_0_, b_1_, …, b_N_) where N is the number of elements. The Golay code exhibits superior pulse-like autocorrelation properties, where the side lobe values of the autocorrelation functions of sequence A and sequence B are exactly opposite and cancel out exactly, resulting in a composite correlation function with a main lobe of 2N and completely absent side lobes, as shown in Equation (3):

(3) A(n)∗A(−n)+B(n)∗B(−n)=2Nδ(n)

**Figure 3:**
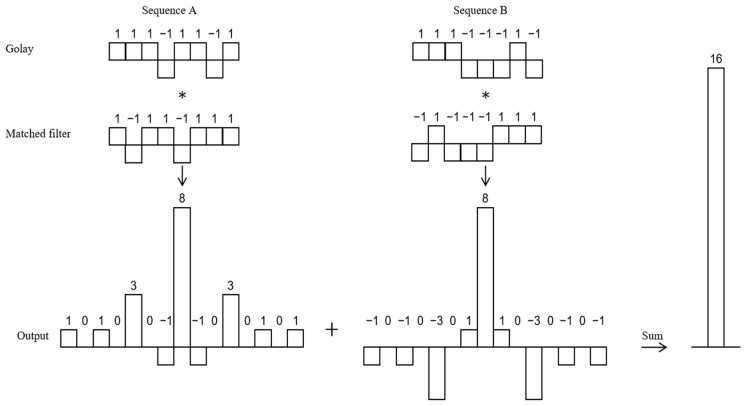
The decoding process for the complementary sequences of the 8-bit Golay code.

Figure 3 depicts the decoding process for the complementary sequences of the eight-bit Golay code.

Reflect the eight-bit sequence A (1, 1, 1, −1, 1, 1, −1, 1) upside down to obtain filter A (1, −1, 1, 1, −1, 1, 1, 1), and reflect the eight-bit sequence B (1, 1, −1, −1, −1, 1, −1) upside down to obtain matched filter B (−1, 1, −1, −1, −1, 1, 1). Then, sequence A is cross-correlated with matched filter A to obtain output A, and sequence B is cross-correlated with matched filter B to obtain output B. Output A and output B add up to a single main lobe of magnitude 16, with all side lobes exactly canceled, and is exactly twice the length of the sequence of 8.

Compared with the traditional single-pulse method, the use of the Golay code to encode the excitation signal can completely suppress the side lobe interference, thereby improving the axial resolution and penetration capability of the signal.

### 2.4. Eight-Bit Golay Code Wide Pulse Signal Modulation

Since the Golay code complementary sequence contains only two elements of “1” and “−1”, it cannot be directly applied in detection, so a wide pulse is usually needed as its carrier. The wide pulse is divided into many short sub-pulses of equal width, and each sub-pulse is modulated with a specific phase.

For example, a wide pulse with a period length of 8T is modulated into a set of eight-bit Golay code time domain signals W(t), as shown in Equation (4):

(4) W(t)=1   0≤t≤8T

**Figure 4:**
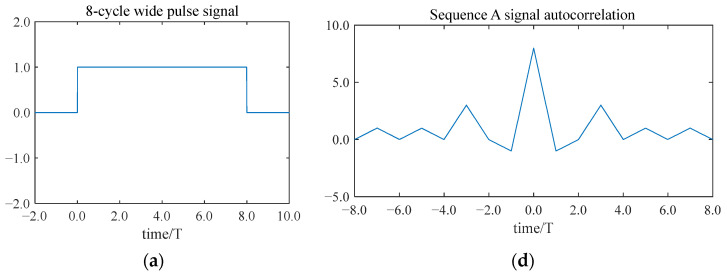

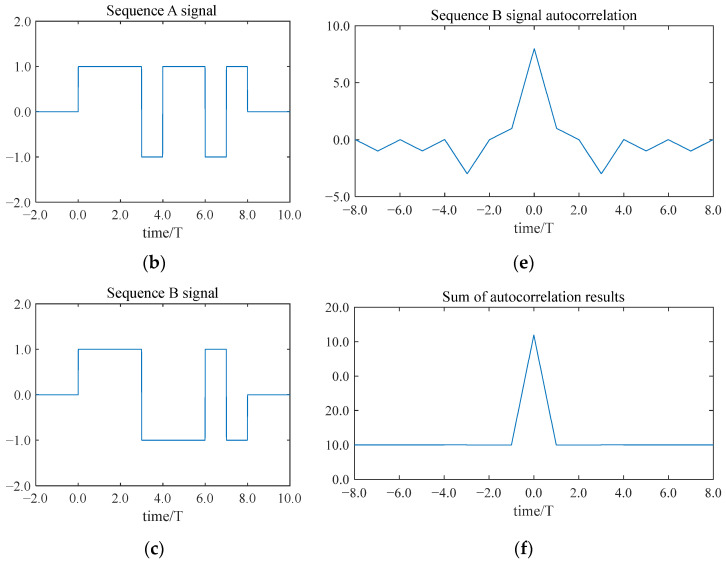
The modulation and decoding of wide pulse signals with 8 cycle lengths:(**a**) A wide pulse signal with a length of 8 cycles; (**b**) the wide pulse sequence A signal; (**c**) the wide pulse sequence B signal; (**d**) the autocorrelation result of the wide pulse sequence A signal; (**e**) the autocorrelation result of the wide pulse sequence B signal; and (**f**) the sum of the autocorrelation results of the signal of sequence A and the autocorrelation results of the signal of sequence B.

Firstly, it is discretized into eight equidistant temporal intervals. Secondly, based on the complementary sequences of the eight-bit Golay code, a phase modulation is performed on each sub-period, where “+1” represents a phase of 0° and “−1” represents a phase of 180°. The eight-bit Golay code complementary sequences are shown in Figure 4.

After modulation, the wide pulse sequence A signal A(t) and the wide pulse sequence B signal B(t) are obtained.

(5) $$A\left(t\right)=\left\{\begin{array}{c} \;\;\;\;\;\;1\;\;\;\;\;\;0\leq t<3T,\;5T\leq t<6T,\;7T\leq t\leq 8T\\ -1\;\;\;\;\;\;\;\;\;3T\leq t<6T,\;\;\;\;\;\;\;\;6T\leq t<7T \end{array}\right.$$

(6) $$B(t)=\left\{\begin{array}{c} \;\;\;1\;\;\;\;\;\;\;\;0\leq t<3T,\;\;\;\;\;\;6T\leq t<7T\\ -1\;\;\;\;\;\;\;\;\;3T\leq t<4T,\;\;\;\;\;\;7T\leq t<8T \end{array}\right.$$

The autocorrelation calculation of A(t) and B(t) is performed separately, and then they are added to obtain the result Y(t).

(7) $$Y(t)=\left\{\begin{array}{c} 16(1-\frac{t}{T})\;\;\;\;\;\;\;\;\;\;\;\;\;\;\;\;\;\;\;\;\;\;\;\;\;\;\;\;\;0\leq t<T\\ 16(1+\frac{t}{T})\;\;\;\;\;\;\;\;\;\;\;\;\;\;\;\;\;\;\;\;\;\;\;-T\leq t<0\\ 0\;\;\;\;\;\;\;\;\;\;\;\;\;\;\;\;\;\;-8T\leq t<-T,\;T\leq t\leq 8T \end{array}\right.$$

Figure 4 illustrates the modulation of a pulse signal with eight periods using the Golay code. Each sub-period is modulated to a specific phase, resulting in a wide sequence A signal and a wide sequence B signal based on the Golay code. The side lobes of the autocorrelation results for both the wide pulse sequence A signal and the sequence B signal are numerically identical but opposite in sign. In addition, they cancel each other out perfectly, leaving a signal with a pronounced main lobe.

### 2.5. Eight-Bit Golay Code Square Wave Pulse Signal Modulation

**Figure 5:**
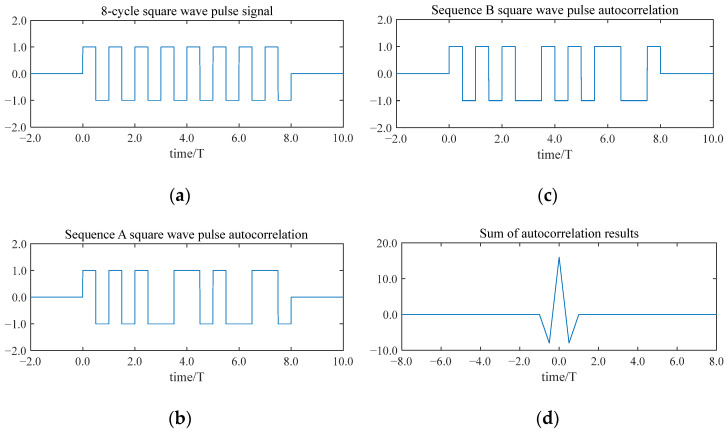
The Golay code complementary sequence square wave pulse modulation and decoding: (**a**) the 8-period square wave pulse signal; (**b**) he sequence A square wave signal obtained by multiplying the square wave pulse signal with the wide pulse sequence A signal; (**c**) the sequence B square wave signal obtained by multiplying the square wave pulse signal with the wide pulse sequence B signal; and (**d**) the sum of the autocorrelation results of the square wave pulse signal A and square wave pulse signal B.

In air-coupled ultrasonic non-destructive testing, a square wave pulse signal is often used as the excitation signal to ensure the ultrasonic transducer reaches the optimal performance. By multiplying a wide pulse sequence A signal and a sequence B signal with square wave pulses (both having the same frequency and period duration), the corresponding square wave pulse sequences’ A and B signals are obtained. Figure 5 depicts a square wave pulse signal with an eight-bit Golay coding modulation.

The decoding results indicate that side-lobe signals are entirely suppressed, leaving only the main-lobe signals. This not only enhances the signal’s penetration capability but also provides superior anti-interference properties. Consequently, this study proposes the adoption of an eight-bit Golay encoded square wave signal to excite the ultrasonic transducer, aiming to achieve a greater penetration and higher accuracy.

### 2.6. Introduction to the Core Components of the Detection System

This system uses two HC75E40TR-1 ultrasonic sensors produced by Hengchuang Sensing Co., Ltd. (Shenzhen, China), with a diameter of 27 mm and a thickness of 24 mm. Its rated frequency is 75 kHz, and the echo sensitivity is 60 dB. The maximum input voltage is 800 Vp-p, which can withstand high-frequency and high-voltage pulse excitation signals, equipping the system with an excellent detection depth in the defect detection of wood. Ultrasonic signals are transmitted through wood and are collected by the receiving transducer, which, according to the principle of the piezoelectric effect, converts the mechanical vibration of sound waves into electrical signals. Then, the direct current component of the electrical signal is isolated by the preamplifier, the signal amplification circuit amplifies the amplitude of the electrical signal, and the band-pass filter filters the noise signal from the electrical signal. Finally, the electrical signal is received by the DAQ acquisition card and enters the host computer through the USB port for decoding and imaging. The preamplifier is an RC blocking circuit that isolates the direct current component in the received signal. The signal amplification circuit is a second-order adjustable amplification circuit, using the AD8421 operational amplifier produced by Analog Devices (Wilmington, MA, USA) as the core of the signal amplification circuit, which has a bandwidth of 10 MHz, a slew rate of 35 V/µs, and a 0.001% (G = 10) stability time of 0.6 µs, and can provide a gain of 10,000 times, which performs excellently in amplifying high-speed signals. The product also has a high common mode rejection ratio (CMRR), which can filter noise signals while extracting effective low-level signals. After the amplification by the amplifying circuit, there is still some noise in the electrical signal, which needs to be further filtered. The band-pass filter circuit uses the LT1567 operational amplifier, a rail-to-rail low-noise high-frequency operational amplifier produced by Analog Devices (Wilmington, MA, USA), as the core of the circuit. By reasonably configuring the resistors and capacitors, a second-order filter circuit with a cutoff frequency as high as 5 MHz can be designed. The center frequency of the band-pass filter circuit designed in this study is 75 kHz, with a −3 dB bandwidth of 14 kHz and a quality factor (Q) of about 2.7, which can effectively suppress the high-frequency and low-frequency noise in the signal, improving the signal quality.

## 3. Experimentation and Analysis

### 3.1. The Construction of the Experimental Platform

**Figure 6:**
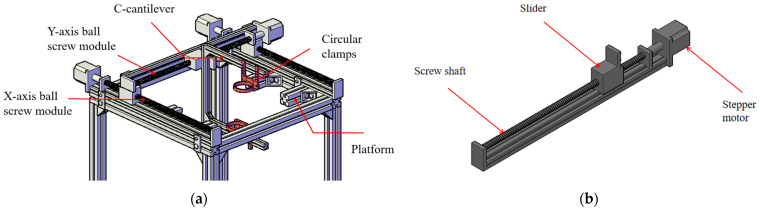
Scanning platform: (**a**) motor system; (**b**) ball screw module.

In order to verify the influence of the improvement of Golay coding technology on the effect of air-coupled ultrasonic testing, a double-axis scanning platform was established. The scanning platform is shown in Figure 6.

It can be seen from the above picture that in the middle of the experimental platform, there is a simple placement platform composed of 4 single-groove aluminum profiles that can hold a 40 cm × 40 cm wooden board. On top of the experimental platform, a ball screw module is installed on the X and Y axes, driven by a stepping motor. A C-cantilever is installed on the Y-axis ball screw module, with upper and lower circular clamps securing air-coupled ultrasonic transducers. The distance between the transducer and specimen is adjusted by precisely repositioning the circular clamps, while the stepper motor controls the transducer’s positioning and scanning intervals through programmable adjustments to its rotation speed and direction.

**Figure 7:**
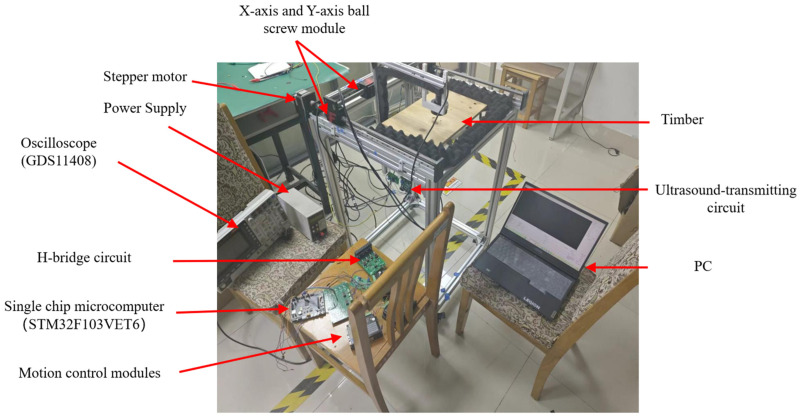
Experimental platform for air-coupled ultrasonic plate flaw detection of Golay code.

Figure 7 shows the photo of the experimental platform, where a 20 cm × 40 cm × 1 cm wooden board is placed on the platform as the testing object. Before the experiment starts, the oscilloscope is used to debug the circuit of each level to confirm that the circuit can work normally. Then, according to the thickness of the sample, the distance between the transducer and the sample is set to 10 cm, and the power supply voltage of the H-bridge circuit is set to 200 V to ensure that the detection system is in the best detection state. Then, on the PC, set the scanning area, scanning speed, and sampling point interval, and input the instructions into the lower computer via serial communication. After the information is verified correctly, the lower computer drives the motor to make the ultrasonic transducer move along the predetermined trajectory until the C-scan is completed. After the C-scan, the collected data are transmitted to the host computer through the CAD card for decoding and imaging.

### 3.2. Introduction to A-Scan and C-Scan

**Figure 8:**
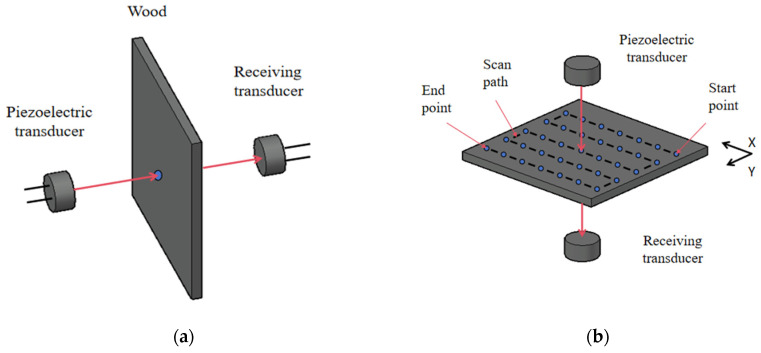
A-scan and C-scan diagram: (**a**) A-scan diagram and (**b**) C-scan diagram.

As shown in Figure 8, the A-scan is a one-dimensional scanning method, and the scanning object is usually a point on the target. The thickness or density of this point is judged according to the flight time or peak height of the ultrasonic signal, which is suitable for the rapid positioning of the defect location.

A C-scan is a two-dimensional scanning mode, and the scanning object is several sampling points arranged in a C-shaped pattern on the sample. During scanning, starting from the starting point, sample all the points in turn along the C-shaped trajectory, and record the X–Y coordinates and signal characteristics of each point. After the C-scan, the signal characteristics of each point are converted into different colored pixels based on their magnitude and then arranged in the corresponding X–Y coordinates to form an RGB image. The resolution of the RGB image is determined by the scanning interval, which is the distance between adjacent sampling points. Finally, the RGB image is interpolated to make the image smoother.

### 3.3. Detect Defects on Pine Boards

**Figure 9:**
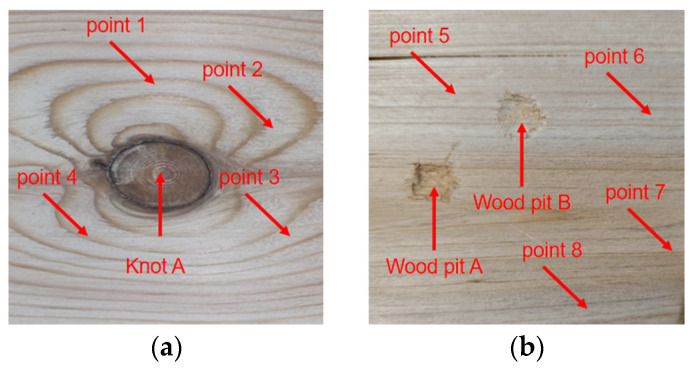
The actual photos of the two pieces of pine wood selected: (**a**) the scanning area of the pine board with knots and (**b**) the scanning area of the pine board with pits.

The most common defects of wood boards are knots and pits, so a pine board with knots and a pine board with pits were selected, as shown in Figure 9. The thickness of the wooden board is 1 cm, the scanning area is 5 cm × 5 cm, the scanning interval is 1 mm, the distance between the two sensors and the wooden board is 10 cm, and the excitation voltage is 200 V.

Firstly, four points were randomly selected in the non-defective areas of the two wooden boards for A-scan testing, and the decoding results of the ultrasonic signals of each point are shown in the following table. It can be seen from Table 1 that the voltage peaks of each sampling point in the non-defective areas change slightly and are close to each other, indicating that the material in the non-defective areas of the pine wood board is relatively uniform.

**Figure 10:**
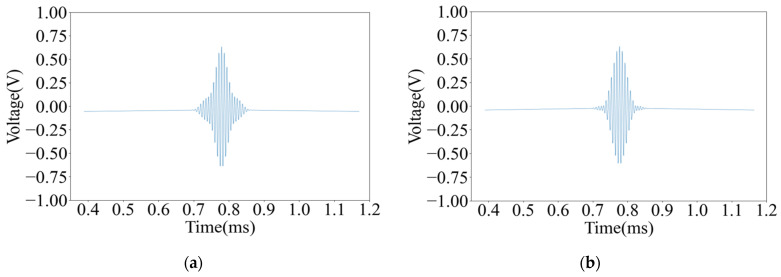
The decoded waveform diagram of signals from point 1 and point 5: (**a**) the waveform diagram of the signal decoding result of point 1 and (**b**) the waveform diagram of the signal decoding result of point 5.

Figure 10 shows the waveform of the signal decoding results between point 1 and point 5. It can be seen from the figure that the received signal is very steep and has an excellent axial resolution.

**Figure 11:**
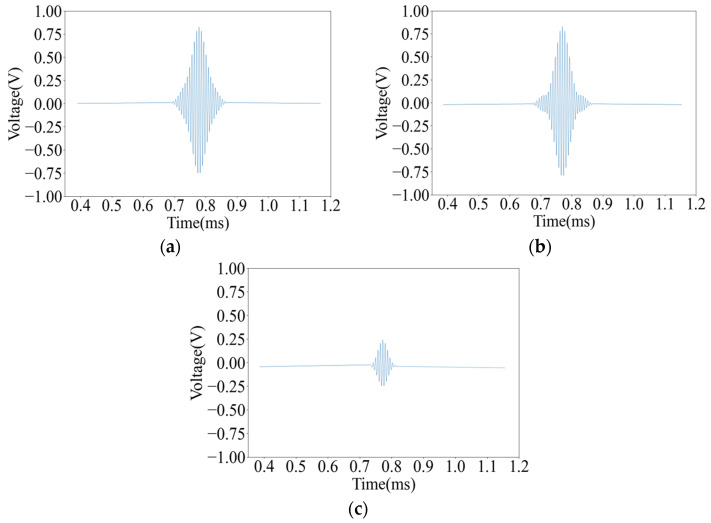
Decoded results of ultrasonic signals at different defect points: (**a**) Pit A decoding result; (**b**) Pit B decoding result; and (**c**) Knot A decoding result.

Thereafter, the two pieces of pine wood board with knots and pits were scanned with an A-scan, respectively, and the decoding results of the ultrasonic signals at each point are shown in Figure 11.

It can be seen from Figure 10 and Figure 11 that the decoding results of the same plank are similar in the non-defective areas, and there is a big difference in the decoding results in the defective areas. The peak voltage of the decoding results in the non-defective areas fluctuates between 0.630 V and 0.654 V; while in the pit areas, the peak voltage of the decoding results is 0.811 V and 0.830 V, respectively, and the peak voltage of the decoding results in the knot area varies more dramatically to 0.251 V.

Although wood is an orthotropic material, the density, moisture content, and elastic modulus of the intact non-defective areas will not undergo a mutation, so the peak voltage of the decoding results of each sampling point in the intact non-defective areas is very close. Because the thickness of the pit areas is less than that of other areas of the pine board, the energy lost by the ultrasound when penetrating this area is less, so the peak voltage of the decoding result in this area is higher. Since the density of the knot area is far greater than other areas of the pine board, the acoustic impedance is higher, resulting in a more severe acoustic impedance mismatch, and the ultrasound loses more energy when penetrating this area. And the knots are mostly formed by branches or dead branches inside the trunk, and the irregular shape will distort the waveform, further reducing the energy of the signal, so the peak voltage of the decoding result in this area is low.

**Figure 12:**
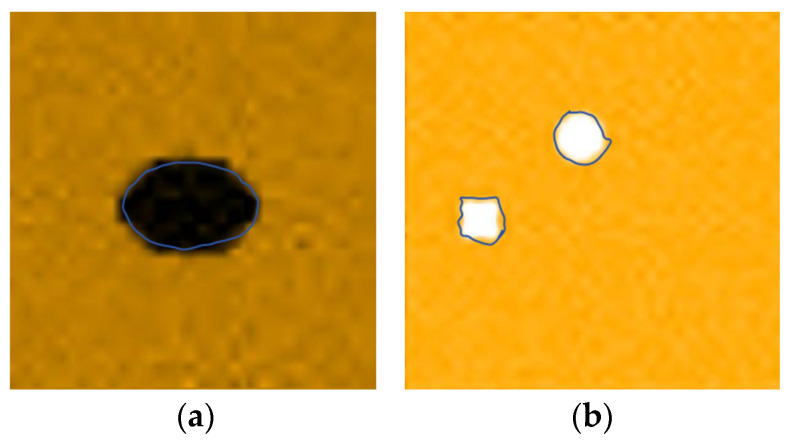
The visualization of the scanning results: (**a**) the imaging of the pine wood with knots and (**b**) the imaging of the pine wood with pits.

Therefore, after the C-scan of the pine wood board, the peak value of the decoding voltage at each point is used as the imaging feature parameter, and the position and type of the defect are judged according to the change in amplitude. The visualization of the scanning results is shown in Figure 12, and the feature parameters of the black part are smaller, indicating the knot area of the pine wood board, and the feature parameters of the white part are higher, representing the pit area of the pine wood board; the blue curve represents the actual contour of the defect area.

**Figure 13:**
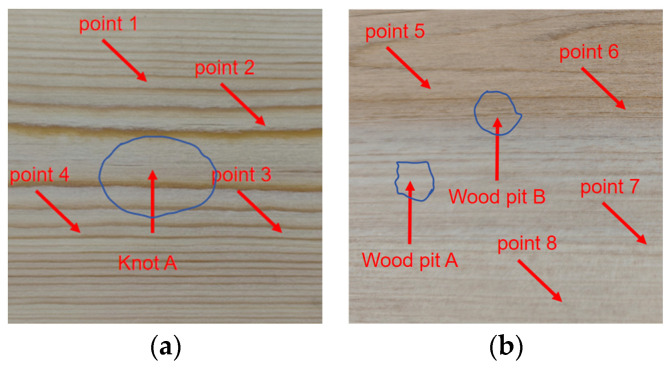
The back of the two pine boards: (**a**) the back of the pine board with knots and (**b**) the back of the pine board with pits.

The inside or back of the wood is also prone to defects. As shown in Figure 13, the original image of the back of the above two boards is shown, and for greater intuitiveness, mirror flipping processing is performed.

A-scans were performed on the back of the wood corresponding to the sampling points, and the results are shown in Table 2.

Similarly to the results of the frontal scanning image, the voltage in the knot area is low, and the voltage in the pit area is high. Since the back of the defect position is flatter than the front, it reduces the absorption of the ultrasonic energy, so the data collected at the same position have a higher voltage. And the voltage amplitude in the non-defective areas hardly changes.

**Figure 14:**
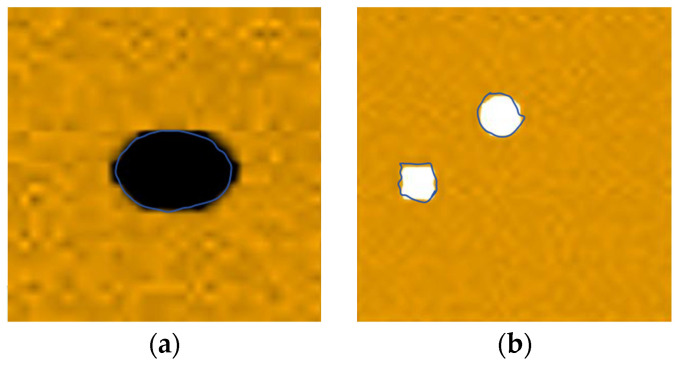
Scanning images of the back of the pine wood board: (**a**) imaging of pine wood with knots and (**b**) imaging of pine wood with pits.

The C-scan imaging diagram is shown in Figure 14, which shows little difference in the imaging effect compared with the frontal scanning diagram, proving that this system has a good detection accuracy for defects inside or on the back of the wood.

### 3.4. Combination Defect Detection of Pine Boards

**Figure 15:**
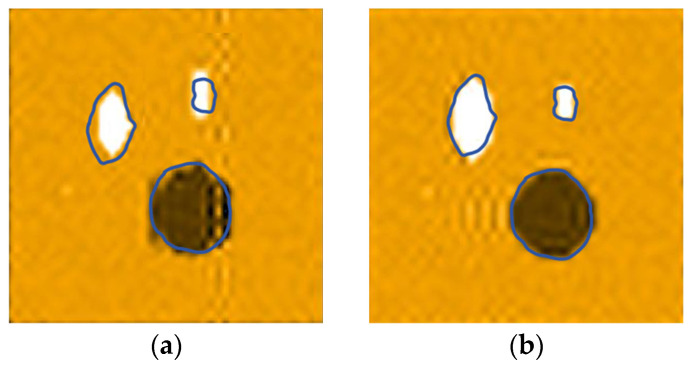

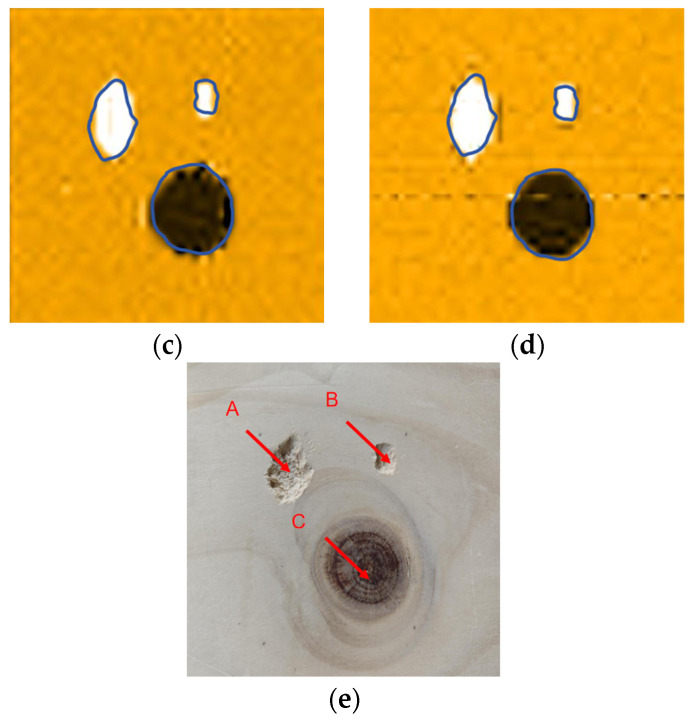
Combination defect detection of pine boards: (**a**) non-encoded signal detection imaging; (**b**) Golay codes encoding signal detection imaging; (**c**) Barker codes encoding signal detection imaging; (**d**) pulse cancelation signal detection imaging; and (**e**) real photo of the pine wood.

In this experiment, ultrasonic signals are excited by using eight-bit pulse signals, eight-bit Golay codes encoding technology, seven-bit Barker codes technology, and the pulse cancelation technique, respectively, and the C-scan experimental results are compared. The scanning object is a piece of pine wood board with a thickness of 1 cm; the size of the scanning area is 6 cm × 6 cm, the interval is 1 mm, the distance between the two transducers and the wood board is 10 cm, and the excitation signal voltage is 200 V. The scanning imaging is shown in Figure 15.

It can be seen from Figure 15 that the imaging effect of using the eight-bit pulse signal is very poor, and the size and shape are quite different from the original image; the imaging effect of the Barker coding technology is general, and the edge of the knot area is not smooth; the imaging effects of both the Golay code encoding technology and pulse cancelation technology are very good—the edges of the image defective area are smooth, and there is a high of overlap with the real defective area.

**Figure 16:**
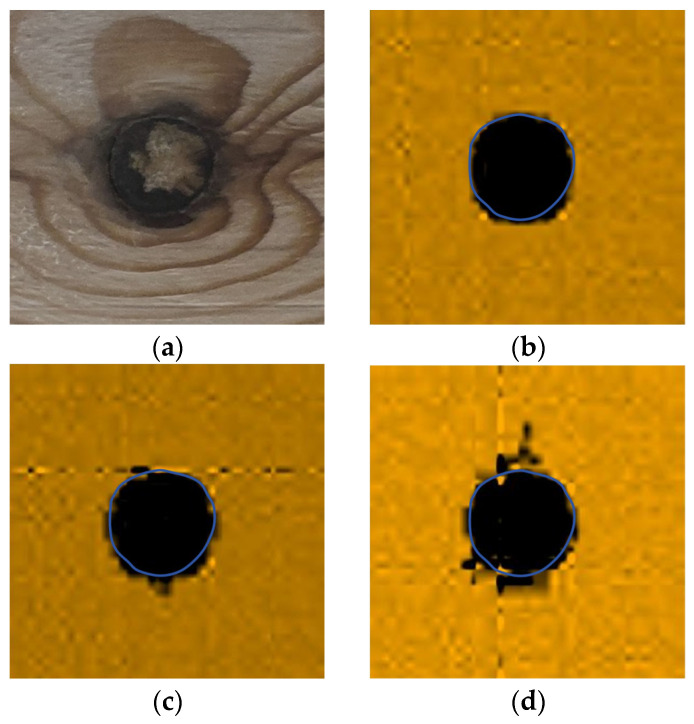
The imaging effect comparison experiment: (**a**) a real photo of the pine wood; (**b**) the Golay codes encoding signal detection imaging; (**c**) the Barker codes encoding signal detection imaging; and (**d**) the pulse cancelation signal detection imaging.

We continued to perform C-scan imaging on a piece of pine board under the same detection conditions. The thickness of the pine wood board is 1.5 cm, and the size of the scanning area is 6 cm × 6 cm. The scanning results are shown in Figure 16.

As can be seen from Figure 16, in the case of increasing the thickness of the wooden plate, the ultrasonic signals excited by the Barker and Golay encoding technologies still have a good penetration capability and can penetrate the wooden plate normally. The ultrasonic signal excited by the pulse elimination technique is misjudged as a knot area because it cannot penetrate some parts of the wooden board, or the energy after penetrating the wooden board is too small.

The Barker code and Golay code are both phase-coding technologies, and they have similar autocorrelation properties, which can effectively suppress side lobes and cause the ultrasonic signal to possess a good penetration ability and axial resolution. However, the Golay code can eliminate side-lobe interference, and compared with Barker, it has a stronger anti-interference ability, and it is easier to extract more accurate signal features from the received signal, so it has a higher detection accuracy for the defective area. Pulse cancelation technology applies a sequence of pulses with opposite phases to the transducer through the method of antiphase pulse cancelation, which shortens the ringing time by energy cancelation, thus improving the axial resolution of the ultrasonic signal and the detection accuracy of the detection system. However, when the thickness of the sample to be measured increases, it is easy to cause a significant decrease in the accuracy of the detection due to the insufficient penetration capability.

The comparison of the real photo of the pine board with the imaging of Golay-coded signals shows that the overlap of the defective areas reaches more than 85%. The type of defect can be judged while identifying the size and position of the defect. A good anti-interference and penetration performance ensures that the system can still maintain a good detection accuracy when the thickness of the sample increases. The test results are consistent with the theory, which proves the effectiveness of the air-coupled ultrasonic technology based on the Golay code in wood detection.

## 4. Conclusions and Future Work

This study uses air-coupled ultrasound technology and excites ultrasonic signals with Golay code pulse signals to perform a non-destructive defect detection on wood. Firstly, the foundational framework of the Golay code encoding methodology is systematically analyzed, with its implementation viability corroborated through rigorous theoretical derivations and simulations. Secondly, the composition of the system, the operation process of the system, the scanning methods of a scanning and c scanning, and the principle of c scanning imaging are introduced in detail. Thirdly, pine wood boards containing natural knot and pit defects were selected as experimental specimens, and an ultrasonic A-scan detection was systematically conducted to compare the difference in the peak voltage in the decoding results between defective areas (areas with knots/pits) and non-defect areas, and we use this as the basis for the C-scan imaging of the wood. Finally, a non-destructive evaluation was performed on pine wood specimens employing three distinct excitation modes—encoded pulse sequence signals, conventional multi-pulse signals, and pulse elimination signals—with a subsequent comparative analysis of their respective imaging performance characteristics. Experimental results show that the use of Golay coding technology can effectively improve the system’s imaging accuracy in the non-defective areas and defective areas of wood. In conclusion, Golay-coded excitation emerges as a viable ultrasonic signal processing strategy that concurrently enhances the penetration depth and axial resolution of the signal, thereby optimizing the inspection depth capability and defect characterization accuracy in wood material evaluation systems. The system demonstrates an exceptional defect localization precision, enabling the rapid characterization of material anomalies in industrial wood processing operations. This capability facilitates optimized cutting vector planning through defect mapping, thereby minimizing the material wastage while improving the production quality and production efficiency of wooden products.

## Figures and Tables

**Table 1:** Peak voltage of 8 sampling points.

Point	1	2	3	4	5	6	7	8
Peak Voltage (V)	0.651	0.636	0.641	0.637	0.654	0.639	0.651	0.648

**Table 2:** Peak voltage of 11 points.

Point	1	2	3	4	5	6	7	8	Knot A	Pit A	Pit B
Peak Voltage (V)	0.648	0.651	0.647	0.646	0.643	0.641	0.648	0.656	0.282	0.845	0.857

## Data Availability

The original contributions presented in this study are included in the article. Further inquiries can be directed to the corresponding author.

## References

[1] Kadunc A. (2013). The incidence of rot in Norway spruce and its influence on the value of trees in Slovenia. Croat. J. For. Eng..

[2] Vega M., Hamilton M.G., Blackburn D.P., McGavin R.L., Baillères H., Potts B.M. (2016). Influence of site, storage and steaming on Eucalyptus nitens log-end splitting. Ann. For. Sci..

[3] Voyer D., Moreau G., Gennaretti F., Bédard S., Havreljuk F., Grondin P., Achim A. (2024). Age and growth reductions increase the proportion of dark heartwood in sugar maple at the northern limit of its range. Forestry.

[4] Sousa H.S., Branco J.M., Lourenço P.B. (2013). Characterization of Cross-Sections from Old Chestnut Beams Weakened by Decay. Int. J. Archit. Herit..

[5] Taskhiri M.S., Hafezi M.H., Harle R., Williams D., Kundu T., Turner P. (2020). Ultrasonic and thermal testing to non-destructively identify internal defects in plantation eucalypts. Comput. Electron. Agric..

[6] Sanabria S.J., Mueller C., Neuenschwander J., Niemz P., Sennhauser U. (2011). Air-coupled Ultrasound as an Accurate and Reproducible Method for Bonding Assessment of Glued timber. Wood Sci. Technol..

[7] Vun R.Y., Wu Q.L., Bhardwaj M.C., Stead G. (2003). Ultrasonic Characterization of Structural Properties of Oriented Strandboard: A Comparison of Direct-contact and Non-contact Methods. Wood Fiber Sci..

[8] Ribeiro P.G., Gonçalez J.C., Gonçalves R., Teles R.F., de Souza F. (2013). Ultrasound Waves for Assessing the Technological Properties of Pinus Caribaea Var Hondurensis and Eucalyptus Grandis Wood. Maderas-Cienc. Tecnol..

[9] Palma S.S.A., Gonçalves R., Trinca A.J., Costa C.P., Reis M.N., Martins G.A. (2018). Interference from Knots, Wave Propagation Direction, and Effect of Juvenile and Reaction Wood on Velocities in Ultrasound tomography. BioResources.

[10] Vössing K.J., Gaal M., Niederleithinger E. (2020). Imaging Wood Defects Using Air Coupled Ferroelectret Ultrasonic Transducers in Reflection Mode. Constr. Build. Mater..

[11] Baradit E., Fuentealba C., Yáñez M. (2021). Elastic Constants of Chilean *Pinus radiata* Using Ultrasound. Maderas-Cienc. Tecnol..

[12] Gonçalves R., Trinca A.J., Ferreira G.C.D. (2011). Effect of coupling media on velocity and attenuation of ultrasonic waves in Brazilian wood. J. Wood Sci..

[13] Woong P.J., Kim D., Im K., Hsu D.K., In-Young Y. (2009). Influence of Resin-Infiltrated Time on Wood Natural Materials Using Conventional/Air-Coupled Ultrasound Waves. J. Korean Soc. Nondestruct..

[14] Hittawe M.M., Sidibé D., Mériaudeau F. A machine vision based approach for timber knots detection. Proceedings of the 12th International Conference on Quality Control by Artificial Vision.

[15] Nurthohari Z., Murti M.A., Setianingsih C. Wood Quality Classification Based on Texture and Fiber Pattern Recognition using HOG Feature and SVM Classifier. Proceedings of the 2019 IEEE International Conference on Internet of Things and Intelligence System (IoTaIS).

[16] Ji M., Zhang W., Han J.K., Miao H., Diao X.L., Wang G.F. (2024). A deep learning-based algorithm for online detection of small target defects in large-size sawn timber. Ind. Crop. Prod.

[17] Lukovic M., Ciernik L., Müller G., Kluser D., Pham T., Burgert I., Schubert M. (2024). Probing the complexity of wood with computer vision: From pixels to properties. J. R. Soc. Interface.

[18] Fang Y.M., Lin L.J., Feng H.L., Lu Z.X., Emms G.W. (2017). Review of the use of air-coupled ultrasonic technologies for nondestructive testing of wood and wood products. Comput. Electron. Agric..

[19] Marhenke T., Neuenschwander J., Furrer R., Zolliker P., Twiefel J., Hasener J., Wallaschek J., Sanabria S.J. (2020). Air-Coupled Ultrasound Time Reversal (ACU-TR) for Subwavelength Nondestructive Imaging. IEEE Trans. Ultrason. Ferroelectr. Freq. Control.

[20] Mori M., Hasegawa M., Yoo J.C., Kang S.G., Matsumura J. (2016). Nondestructive evaluation of bending strength of wood with artificial holes by employing air-coupled ultrasonics. Constr. Build. Mater..

[21] Kazys R., Sliteris R., Sestoke J., Vladisauskas A. (2015). Air-Coupled Ultrasonic Transducers Based on an Application of the PMN-32%PT Single Crystals. Ferroelectrics.

[22] Zhang H.D., Xu L., Zhou G.P., Zhao L., Yu J.W., Ye Q.Y. (2023). Research on vibration characteristics of the longitudinal-radialcomposite piezoelectric ultrasonic transducer. Appl. Acoust..

[23] Zhang Q., Luo C.Z., Wang R.X., Guo X.H. (2024). Investigation on Characteristics of a MXenes-Based Air-Coupled Capacitive Ultrasonic Transducer Array. IEEE Sens. J..

[24] Yu Y.Y., Cao X.W., Pun S.H., Mak P.U., Vai M.I. (2016). Output pressure enhancement of CMUTs by using multiple Helmholtz resonance apertures. Electron. Lett..

[25] Fang H.J., Chen Y., Wong C.M., Qiu W.B., Chan H.L.W., Dai J.Y., Li Q., Yan Q.F. (2016). Anodic aluminum oxide-epoxy composite acoustic matching layers for ultrasonic transducer application. Ultrasonics.

[26] Kusano Y., Wang Q., Rudy R.Q., Polcawich R.G., Horsley D.A. (2017). Wideband Air-Coupled Pzt Piezoelectric Micromachined Ultrasonic Transducer Through Dc Bias Tuning. Proceedings of the 30th IEEE International Conference on Micro Electro Mechanical Systems (MEMS).

[27] Kusano Y., Wang Q., Luo G.-L., Lu Y., Rudy R.Q., Polcawich R.G., Horsley D.A. (2018). Effects of DC Bias Tuning on Air-Coupled PZT Piezoelectric Micromachined Ultrasonic Transducers. J. Microelectromech. Syst..

[28] Liu X.X., Chen X.Y., Le X.H., Wang Y., Wu C.J., Xie J. (2018). Reducing Ring-Down Time of pMUTs with Phase Shift of Driving Waveform. Sens. Actuator A-Phys..

[29] Yang H.C., Cannata J., Williams J., Shung K.K. A study of 1–3 pseudo-random pillar piezocomposites for ultrasound transducers. Proceedings of the IEEE International Ultrasonics Symposium (IUS).

[30] Wu Z.P., Liu W.J., Tong Z.H., Cai Y., Sun C.L., Lou L. (2022). Tuning Characteristics of AlN-Based Piezoelectric Micromachined Ultrasonic Transducers Using DC Bias Voltage. IEEE Trans. Electron Devices.

[31] Wu Z.P., Liu W.J., Tong Z.H., Zhang S.S., Gu Y.D., Wu G.Q., Tovstopyat A., Sun C.L., Lou L. (2021). A Novel Transfer Function Based Ring-Down Suppression System for PMUTs. Sensors.

[32] Trots I., Nowicki A., Lewandowski M., Secomski W., Litniewski J. (2008). Double pulse transmission—Signal-to-noise ratio improvement in ultrasound imaging. Arch. Acoust..

[33] Gong P., Song P.F., Chen S.G. Hadamard encoded multi-pulses for contrast enhanced ultrasound imaging. Proceedings of the IEEE International Ultrasonics Symposium (IUS).

[34] Hu C.H., Liu R.B., Zhou Q.F., Yen J., Shung K.K. (2006). Coded excitation using biphase-coded pulse with mismatched filters for high-frequency ultrasound imaging. Ultrasonics.

[35] Diao X.F., Zhu J., He X.N., Chen X., Zhang X.Y., Chen S.P., Liu W.X. (2017). An ultrasound transient elastography system with coded excitation. Biomed. Eng. Online.

[36] Wang M.Q., Cong S., Zhang S. Pseudo Chirp-Barker-Golay Coded Excitation in Ultrasound Imaging. Proceedings of the 30th Chinese Control and Decision Conference (CCDC).

[37] Garcia-Rodriguez M., Yañez Y., Garcia-Hernandez M.J., Salazar J., Turo A., Chavez J.A. (2010). Application of Golay codes to improve the dynamic range in ultrasonic Lamb waves air-coupled systems. NDT&E Int..

[38] Tang J.Y., Zhu W.J., Qiu X.L., Song A.L., Xiang Y.X., Xuan F.Z. (2021). Non-contact phase coded excitation of ultrasonic Lamb wave for blind hole inspection. Ultrasonics.

[39] Yang Y., Wang P., Jia Y.L., Jing L.X., Shi Y., Sheng H.W., Jiang Y., Liu R.B., Xu Y.H., Li X. (2024). Rail fracture monitoring based on ultrasonic-guided wave technology with multivariate coded excitation. Ultrasonics.

[40] Germano E., Tabatabaeipour M., Mohseni E., Lines D., Macleod C.N., Lam K.H., Hughes D., Trodden H., Gachagan A. (2025). Application of Golay-based total focusing method using a high-frequency, lead-free, flexible ultrasonic array for inspection of thick non-planar industrial components. NDT&E Int..

